# CT-Based Evaluation of Hounsfield Units—A Novel Screening Tool for Undiagnosed Osteoporosis in Patients with Fragility Fractures of the Pelvis

**DOI:** 10.3390/jcm14103346

**Published:** 2025-05-12

**Authors:** Johannes Gleich, Elisabeth Steiner, Christian Ehrnthaller, Nikolaus Degen, Christopher Lampert, Wolfgang Böcker, Carl Neuerburg, Christoph Linhart

**Affiliations:** Department of Orthopaedics and Trauma Surgery, Musculoskeletal University Center Munich (MUM), LMU University Hospital, LMU Munich, 80336 Munich, Germany; johannes.gleich@med.uni-muenchen.de (J.G.); elisabeth.steiner@med.uni-muenchen.de (E.S.); christian.ehrnthaller@med.uni-muenchen.de (C.E.); nikolaus.degen@gmx.de (N.D.); christopher.lampert@med.uni-muenchen.de (C.L.); wolfgang.boecker@med.uni-muenchen.de (W.B.); carl.neuerburg@med.uni-muenchen.de (C.N.)

**Keywords:** pelvic insufficiency fracture, Hounsfield units, osteoporosis, orthogeriatric

## Abstract

**Background**: This institutional, register-based analysis aimed to evaluate the feasibility of using CT-based sacral Hounsfield units (HUs) for assessing bone density in pelvic fragility fractures and to explore their potential correlation with DEXA measurements and osteological laboratory diagnostics. **Methods**: Patients aged > 80 years, admitted between 2003 and 2019 with pelvic ring fractures, were analyzed in this retrospective single-center study. CT scans were evaluated according to the classification of fragility fractures of the pelvis (FFPs), which guided treatment decisions (conservative or surgical). The diagnosis of a fragility fracture was based on both fracture morphology and patient history, including the presence of low-energy trauma. Bone health was assessed using standardized laboratory diagnostics including serum calcium, phosphate, alkaline phosphatase, and 25(OH)-vitamin D, in addition to DEXA scans and CT-derived Hounsfield units. Vitamin D levels and bone density evaluations were analyzed to identify possible correlations among these factors and with fracture patterns. **Results**: A total of 456 patients (mean age 87.3 years, 79.6% female) were included. The CT-based FFP classification identified Type II as the most common fracture type (66.7%). Conservative treatment was the predominant approach (84.9%). Serum 25(OH)-vitamin D deficiency was observed in 62.7% of the patients, while osteopenia and osteoporosis were found in 34.3% and 46.5% of cases, respectively. HU values at S1 showed significant correlation with femoral neck T-scores, highlighting the utility of CT scans for bone density assessment. **Conclusions**: This study emphasizes the complementary roles of CT-derived HU values and DEXA T-scores in evaluating bone quality and fracture severity in geriatric patients with FFP. While DEXA remains the gold standard, CT imaging offers valuable early insights, supporting the timely initiation of osteoporosis therapy. Given the high prevalence of fragility fractures in this age group, early CT-based screening may facilitate earlier initiation of osteoporosis-specific therapy, including anabolic agents where indicated. Further research is needed to explore the relationships between vitamin D levels, bone density assessments, and fracture types.

## 1. Introduction

Osteoporosis is defined as a systemic skeletal disease characterized by reduced bone mineral density (BMD) and deteriorated microarchitecture of the bone tissue. Geriatric patients are especially at risk of suffering osteoporotic fractures and thereafter become bedridden with immobility-related complications [1]. The most common osteoporotic fractures occur at the pelvis/hip, the spine, and upper extremities [1,2,3]. In particular, the incidence of fractures of the pelvic ring shows a positive correlation with age (mostly around 80 years) and morbidity [4,5]. A simple fall of an elder can result in a fragility fracture, which may be defined as a pathological fracture resulting from minimal trauma (a mechanical force that would not ordinarily cause a fracture). An association of fragility fractures of the pelvis (FFPs) with reduced bone quality has already been shown [6,7]. Due to increased life expectancy and the subsequent demographic shift, a sharp rise in the number of fragility fractures is expected. An accurate diagnosis, classification, and well-chosen treatment is crucial to obtain mobility and independence in activities of daily living in orthogeriatric patients. The AO/OTA classification (Arbeitsgemeinschaft für Osteosynthesefragen/Orthopaedic Trauma Association) is used worldwide for classifying fractures and does not consider the differing genesis of pelvic ring fractures in the elderly. Therefore, Rommens et al. proposed a new classification for FFPs in 2018, which considers low-energy trauma with subsequent special fracture patterns, as well as the fragility aspect of geriatric patients [7]. Based on this classification, new treatment patterns were developed. An important aspect after the initial treatment, independent whether conservative or operative, is the so-called secondary fracture prevention. A prompt evaluation of bone quality to treat eventually reduced bone mineral density and prevent further fractures is essential, but this often missed during in-hospital treatment [8]. The gold standard for measuring BMD at the hip or lumbar spine is dual-energy X-ray absorptiometry (DEXA) [9]. Often, only a delayed implementation of this examination is possible or it is completely missed [10]. If already existing data could be used instead, timely assessment and following treatment of reduced BMD would be possible. Medical imaging, particularly computed tomography (CT), is often performed for accurate fracture diagnosis at admission to a hospital [11]. In modern CT with automatic exposure control (AEC), a homogenous energy spectrum can be generated, whereby the measurement of tissue density in Hounsfield units (HUs) is possible without additional costs or radiation exposure. The evaluation of HU values for assessing bone density in different regions of the skeleton has already been investigated, as well as its correlation with other parameters of bone mineral density [12,13,14,15].

The aim of this institutional, register-based analysis was to evaluate the applicability of CT-based sacral Hounsfield units in the assessment of bone density in fragility fractures of the pelvis and its potential correlation with DEXA values, osteological laboratory diagnostics, and different fracture patterns.

## 2. Results

In total, 456 patients with a mean age of 87.3 (±4.5) years were included for final analysis, and 79.6% were female. Diagnosis and classification of the FFP was performed in 100% of the cases by CT, and FFP Type II was most frequently (66.7%) observed (Table 1). Conservative treatment was carried out in most of the patients (84.9%). If treated surgically, there was a significantly increasing proportion in unstable fracture patterns (Table 2). An osteological basic assessment was conducted in 37.1% of the patients regarding the measurement of serum 25(OH)-vitamin D levels and in 21.7% regarding a DEXA scan (see Table 1). The number of patients with CTs suitable for HU analysis was 251 (55.0%). Reasons for exclusion included insufficient image resolution, metal artifacts, or missing axial/sagittal reconstructions. A total of 62.7% of the evaluated patients presented a vitamin D deficiency, and 34.3%/46.5% had underlying osteopenia/osteoporosis (based on DEXA results). In total, 14.7% of the patients demonstrated with anti-osteoporosis therapy based on previously diagnosed osteoporosis. Mean serum 25(OH)-vitamin D levels varied significantly between the different FFP types, with the highest mean in FFP type IV fractures (Table 3). There was no significant correlation between serum 25(OH)-vitamin D levels and FFP patterns. T-scores (measured by DEXA scans of lumbar vertebrae and femoral neck) showed no significant differences between the groups but a trend of increasingly negative T-scores in higher grades of instability. Female patients showed elevated alkaline phosphate (ALP) across all types of FFP, with a trend toward higher alkaline phosphate levels in more complex types of FFP (Table 3). Male patients demonstrated increased ALP levels in FFP type I and IV without significant differences between the groups. Serum calcium, as well as serum phosphate levels, were normal, without significant intergroup differences.

**Figure 1:**
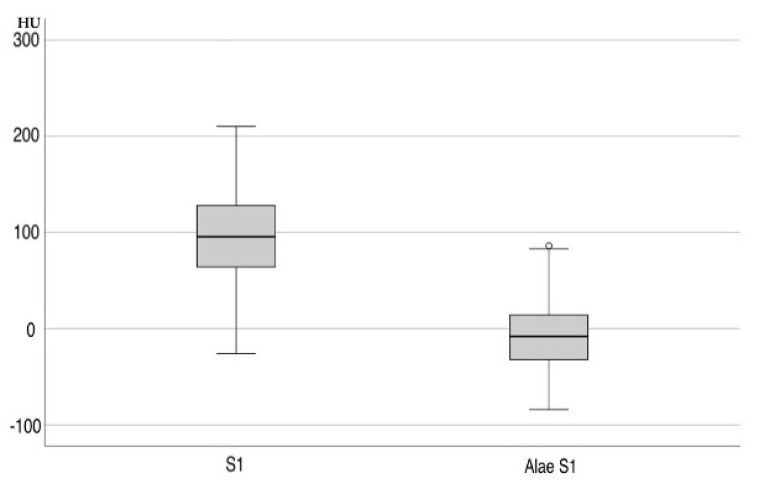
Distribution of Hounsfield units in S1 and alae S1.

**Figure 2:**
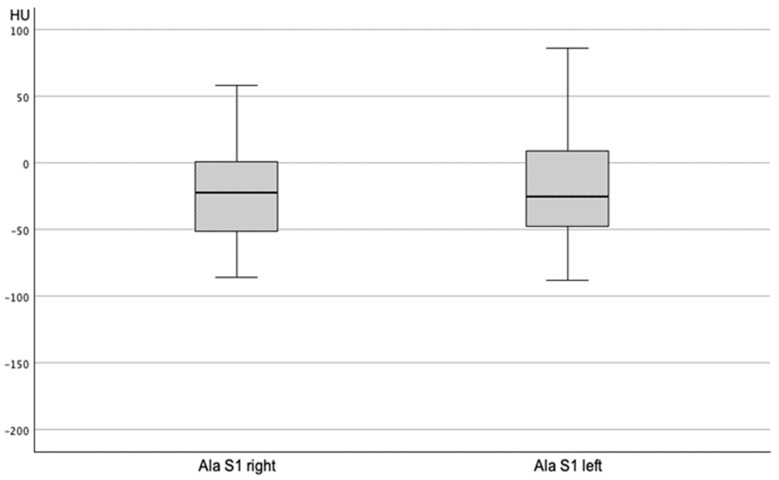
Distribution of Hounsfield units in unfractured Ala S1 right and left.

**Figure 3:**
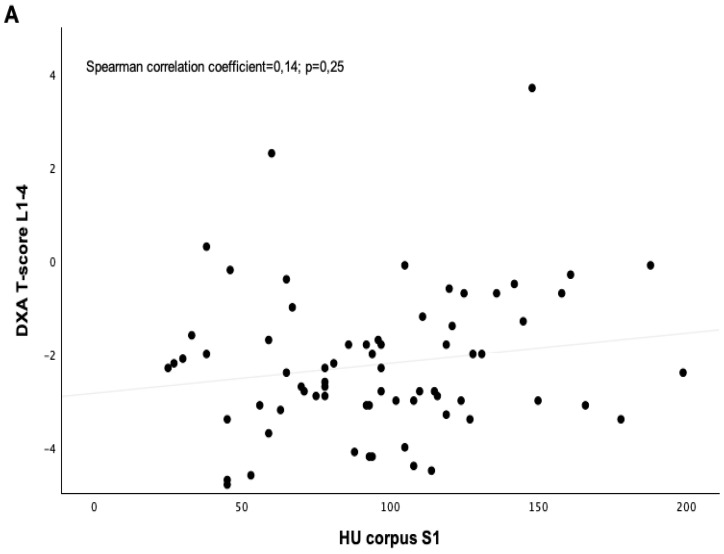

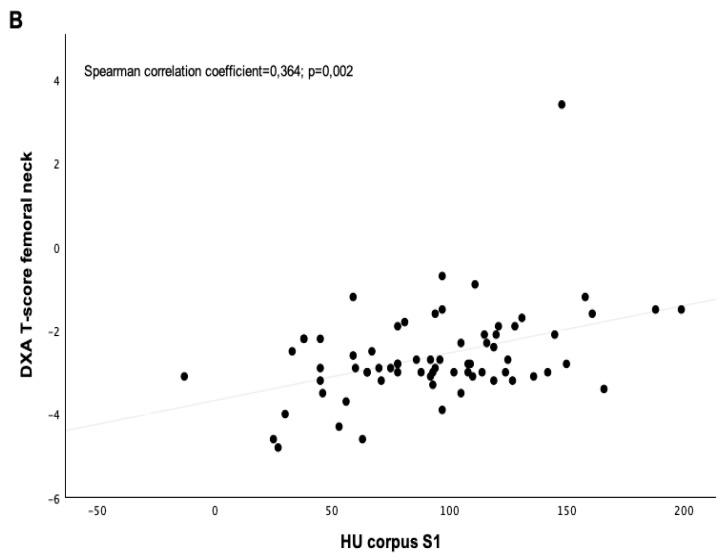
(**A**) Correlation between lumbar T-scores and sacral HUs; R2 linear = 0.028; (**B**) correlation between femoral neck T-scores and sacral HUs; R2 linear = 0.174 (correlation analysis with Spearman correlation coefficient).

A CT-based assessment of Hounsfield units was feasible in 55% of the patients (*n* = 251). Significant differences were found among the sacral regions (*p* < 0.001; Table 4), with HU values at the S1 body (97.90 ± 3.17) and substantially lower values in the alae (–6.70 ± 3.15) (Figure 1). HU values in sagittal and axial planes at the S1 body showed a high internal correlation (*p* < 0.001). Women exhibited significantly lower axial HU values at the S1 level (84.75 ± 53.63) than men (115.86 ± 53.94; *p* < 0.001). Side-specific differences were also present, with the right side –22.66 ± 3.24 vs. left side –18.43 ± 3.34 (Figure 2). HU values at S1 significantly correlated with femoral neck T-scores (*p* < 0.002) but not with lumbar T-scores (*p* = 0.25) (Figure 3A,B). In vitamin D-deficient patients, lower HU values and more negative T-scores were observed at the femoral neck. In contrast, lumbar spine T-scores appeared paradoxically better (Table 5, Table 6 and Table 7).

Among patients stratified by DEXA-based bone density, the highest HU values were found in those with normal BMD (113.33 ± 44.95), followed by osteoporosis (91.27 ± 38.14) and the lowest in patients with osteopenia (87.85 ± 42.06).

## 3. Discussion

This study examines diagnostic and therapeutic approaches, alongside osteological parameters, in 456 elderly patients with fragility fractures of the pelvis (FFPs). The cohort had a mean age of 87.3 years with a predominance of female patients (79.6%). The findings provide key insights into the relationship between vitamin D levels, bone density, and fracture stability, as well as the frequency of conservative and surgical treatments.

Descriptive analysis revealed a strong correlation between HU values measured in the axial and sagittal planes (*p* < 0.001), in line with previous studies by Zou et al. [16]. Similarly to prior findings, HU values in the sagittal plane were consistently lower than those in the axial plane. Region-specific differences were also evident, with significantly lower HU values in the sacral alae compared to the S1 body (*p* < 0.001), corroborating earlier research [12,16,17,18,19]. The significant lower HU values in female patients in the S1 body than in male patients correspond to findings by Wagner et al. [19]. The assessment of significantly higher Hounsfield units (*p* < 0.001) in the S1 alae on the fractured side compared to lower values on the non-fractured side contradicts Schönenberg et al., who reported lower HU values in fracture groups than in non-fractured controls [18]. The increased bone density on the fractured side may be due to the timing of the CT scan, which was not always performed immediately after admission/trauma. Additionally, the chosen region of interest (ROI), including fracture lines with associated remodeling processes, may explain this finding. The hypothesis that the lowest HU values would be associated with the highest FFP group could not be confirmed.

In comparison to the study by Citek et al., which evaluated 45 patients with a mean age of 63.3 years using internally calibrated qCT-based vBMD measurements alongside DXA scans, our cohort consisted of a substantially older population (mean age 87.3 years) with a higher proportion of female patients and a specific focus on fragility fractures of the pelvis. Despite methodological differences—including variations in CT calibration protocols and anatomical focus (lumbar vertebrae vs. sacrum)—both studies demonstrated meaningful correlations between CT-based bone density parameters and DXA-derived T-scores [20].

Notably, our data also showed that patients with normal bone mineral density exhibited the highest sacral HU values, while those with osteopenia had the lowest—mirroring the graded pattern observed in calibrated vBMD categories in the comparative study [20]. However, unlike the referenced study, we found that lumbar spine T-scores did not significantly correlate with sacral HU values, suggesting potential regional variability in BMD sensitivity or structural compensation mechanisms in advanced age.

Furthermore, while the previous study identified novel vBMD thresholds for improved osteoporosis detection through ROC analysis, our findings underscore the potential utility of HU-based assessments in routine trauma imaging, particularly in settings where DXA is unavailable or underutilized. Collectively, these results highlight the growing value of CT-derived bone metrics in complementing conventional diagnostics, especially in geriatric populations with pelvic fragility fractures.

Davidson et al. (2023) [21] conducted a retrospective study involving 93 patients who underwent both DXA and CT imaging within six months. Their analysis demonstrated that DXA overestimated bone mineral density (BMD) compared to Hounsfield unit (HU)-based CT measurements in 97–100% of cases. Depending on the HU threshold applied, the discrepancy rate between DXA and CT assessments ranged from 40% to 54%. These findings suggest that DXA frequently yields higher BMD values than CT, which is particularly relevant when evaluating bone quality in contexts such as spinal surgery. CT-derived HU measurements may thus serve as a valuable adjunct to DXA for a more accurate assessment of bone integrity.

No significant correlation was found between Hounsfield units and FFP types, a result also described by Graul et al. [22]. The heterogeneity of the groups should be considered when interpreting these results, as it may affect the validity of the findings. The measurement of Hounsfield units, particularly the correlation analysis between HUs and T-scores, is increasingly featured in osteological journals [17,18]. CT-based classification of FFPs revealed Type II fractures as the most common, accounting for 66.7% of cases. This aligns with the literature indicating that more unstable fractures are prevalent in older patients. Conservative treatment was employed in 84.9% of cases, likely due to frequent comorbidities and limited physical resilience in this population. Surgical interventions accounted for only 15.1% of cases, with a significant increase observed in more unstable fracture patterns. This suggests that fracture instability is the primary determinant of invasive treatment, consistent with other studies [23,24]. Evaluating the potential effects of conservative treatment on kyphotic deformity and sagittal alignment requires careful risk–benefit assessment, especially in elderly or frail patients. Persistent kyphosis may lead to postural imbalance, reduced mobility, and impaired quality of life. Nevertheless, conservative management offers key advantages, particularly the avoidance of general anesthesia. Given the well-known risks of anesthesia-related complications—such as delirium, immobility, and functional decline—in geriatric patients, careful patient selection is critical. While early surgical stabilization may be necessary to prevent deformity progression in cases of structural instability, non-operative approaches remain essential for minimizing perioperative risks in this vulnerable population.

Although no significant correlation between osteoporosis severity, as assessed by HU values or DXA T-scores, and FFP types was observed, this finding may reflect a threshold versus occurrence effect. It is possible that once a critical level of bone fragility is reached, the specific type or morphology of pelvic fractures is more influenced by additional factors such as the trauma mechanism, patient comorbidities, or local anatomical variations, rather than by further decreases in bone mineral density. This suggests that bone quality, while crucial for the initial risk of fragility fracture occurrence, may have a lesser impact on determining the exact fracture type within the FFP classification once a threshold of structural weakness is exceeded. Future studies should explore this concept by incorporating biomechanical analyses and longitudinal designs to better differentiate the contributions of systemic bone fragility and local mechanical forces to fracture morphology.

A significant finding was the high prevalence of vitamin D deficiency (62.7%) and osteopenia/osteoporosis, diagnosed in 34.3% and 46.5% of patients, respectively. This highlights the need for preventive measures such as adequate vitamin D supplementation and bone density monitoring in geriatric patients. While vitamin D levels varied across different fracture types, no significant correlation was found between vitamin D levels and specific fracture types. However, vitamin D-deficient patients exhibited a trend toward lower HU values in the sacral region, suggesting a decline in bone quality. A link between vitamin D deficiency and reduced bone mineral density has already been identified in previous studies [8,25]. Bone density assessment via T-scores from DEXA scans showed no significant differences between fracture groups. Nonetheless, a trend toward more negative T-scores in complex fractures suggests that decreasing bone density may contribute to fracture instability. HU values derived from CT scans provided additional insights into bone quality. Significant differences in HU values at the S1 level of the sacrum were observed between regions, with men displaying higher HU values than women. This points to potential gender-specific differences in bone structure and density. Unlike T-scores, no clear correlation between HU values and fracture types was found, indicating that HU values may be less sensitive indicators of bone density than DEXA-based T-scores. Notably, HU values at the S1 sacral body correlated significantly with femoral neck T-scores, suggesting that HU values could serve as an alternative method for assessing bone density. This correlation was not observed for the lumbar spine, indicating possible regional differences in bone quality. The bone density at the S1 level assessed by HUs showed no significant correlation with lumbar T-scores, which aligns with previous data [17,18]. Flanigan et al. also described a higher correlation between bone density measurements by DEXA at the femoral neck and sacral HUs [17]. This supports the notion that CT-HU analysis can serve as a surrogate marker for bone density, particularly in the absence of DEXA.

Davidson et al. even demonstrated in a retrospective study involving 93 patients that DEXA overestimated bone mineral density compared to Hounsfield unit-based CT measurements in 97–100% of their cases. Depending on the HU threshold applied, the discrepancy rate between DXA and CT assessments ranged from 40% to 54%. These findings suggest that DXA frequently yields higher BMD values than CT [21].

CT imaging is often already performed during acute care. If HU analysis is validated and automated, it could replace or supplement DEXA in initial assessment. This would enable earlier therapy, including the use of anabolic agents, as recommended when a fragility fracture is present (per clinical guidelines).

This study has several limitations. Its retrospective, registry-based design limits control for confounding variables and introduces potential selection bias. Only 55% of patients had CT scans of sufficient quality for Hounsfield unit (HU) analysis, primarily due to issues with image resolution, metal artifacts, or incomplete reconstructions, which may reduce the generalizability of the findings. DEXA scans or full laboratory assessments were available in a minority of cases, limiting some correlation analyses. Additionally, the cross-sectional design prevents causal inference regarding bone quality and fracture morphology.

Nevertheless, notable strengths include the large, well-defined geriatric cohort and the opportunistic use of existing CT data for bone quality assessment without additional radiation exposure. The demonstrated correlations between HU values, T-scores, and fracture characteristics provide meaningful clinical insights. Given that CT scans are routinely performed for FFP diagnosis, integrating HU assessments could enable the prompt initiation of osteoporosis therapy without the need for additional diagnostic procedures such as DEXA scans, which are not universally available. In Germany, DEXA scans cost between EUR 30 and EUR 60, while CT scans range from EUR 200 to EUR 400. If CT-derived HU values are validated as reliable proxies for bone density, they could reduce the need for separate DEXA testing, offering a cost-effective alternative. This integration could streamline patient management and alleviate financial burdens on healthcare systems, particularly in settings lacking DEXA availability. While DEXA remains the gold standard for osteoporosis diagnosis, incorporating HU assessments into routine fracture evaluation could optimize fragility fracture management in osteoporotic patients, especially where DEXA is inaccessible.

## 4. Materials and Methods

In this retrospective single-center study, all patients aged > 80 years who were admitted to a level one trauma center with a fracture of the pelvic ring were prospectively included from 1 January 2003 to 31 December 2019 into an institutional registry. Approval was obtained from the local ethics committee (reg. no. 518-18). Fragility fractures were defined based on a combination of clinical and radiological criteria. Clinically, classification was guided by the presence of a low-energy trauma mechanism—typically a fall from standing height—which is characteristic in elderly or osteoporotic patients. Additionally, fracture morphology was assessed using computed tomography (CT) imaging and categorized according to the fragility fracture of the pelvis (FFP) classification system introduced by Rommens et al. This classification allows for a detailed evaluation of pelvic fragility fractures, considering fracture location, stability, and biomechanical loading characteristics. Exclusion criteria were defined as follows: any type of cancer, spontaneous fractures without preceding trauma, and high-energy trauma. The main reasons for exclusion from the Hounsfield unit (HU) analysis were insufficient image quality due to low CT resolution, lack of multiplanar reconstructions—particularly in the sagittal plane—and the presence of metal artifacts, which impaired accurate bone density assessment.

**Figure 4:**
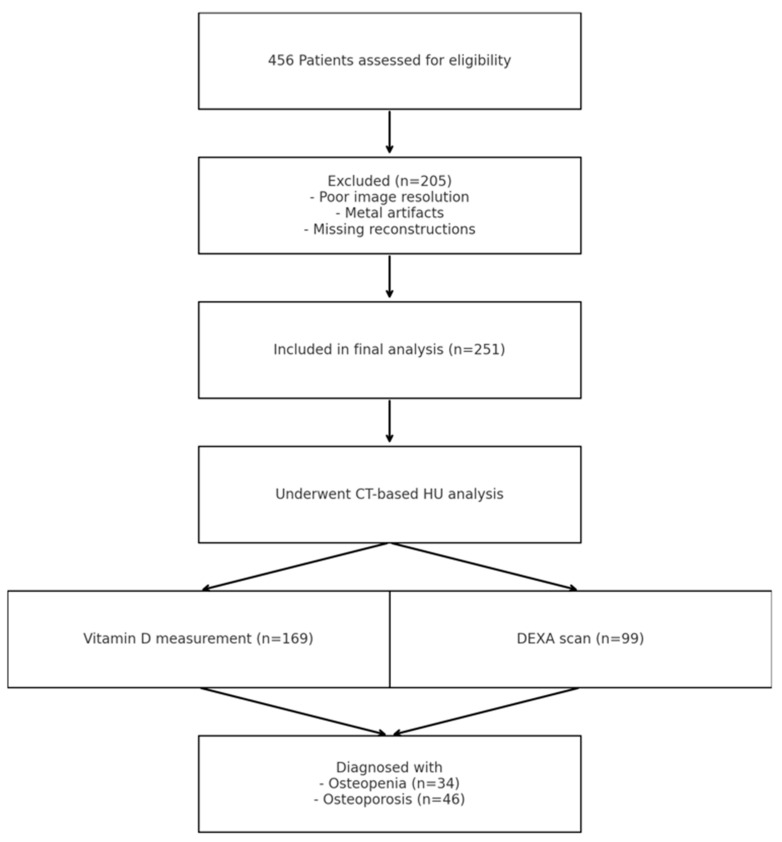
Flowchart illustrating patient selection.

All patients or their legal representative gave written informed consent for inclusion. The study was conducted in accordance with the Declaration of Helsinki (Figure 4).

**Figure 5:**
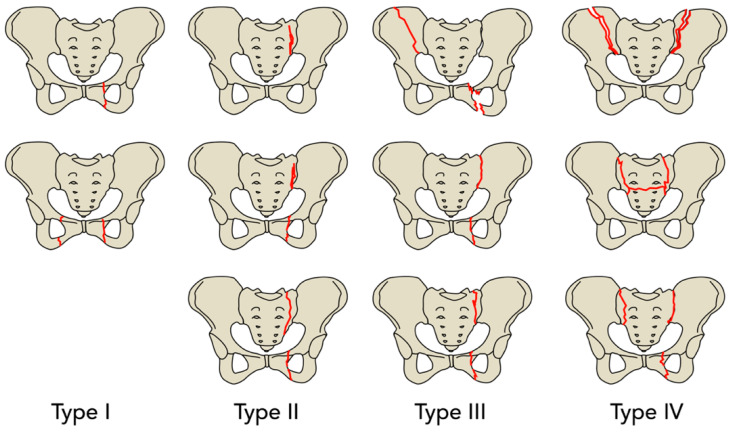
Classification of fragility fractures of the pelvis according to Rommens et al. FFP type I: Only anterior pelvic ring injury. FFP type II: Non-displaced posterior pelvic ring injury. FFP type III: Displaced unilateral posterior pelvic ring injury (in type II and III, usually additional affection of the anterior pelvic ring). FFP type IV: Displaced bilateral posterior pelvic ring injury.

Fracture classification was CT-based, according to the previously mentioned FFP classification by Rommens et al. This classification ranges from FFP Type I to FFP Type IV lesions and is based on the degree of instability (Type I: limited instability, Type IV: highest instability; see Figure 2). Osteoporotic, fatigue, and insufficiency fractures are summarized [11] (Figure 5). If conservative treatment was indicated, standardized pain medication following WHO (World Health Organization) treatment guidelines was administered, and mobilization by physiotherapists started as soon as possible with the goal of full weight-bearing of the injured site; if mobilization was impossible after 3 to 5 days from admission, operative treatment was indicated. Surgery was performed under general anesthesia by trauma specialists according to the AO principles of fracture management and the recommendations for the treatment of FFP by Rommens et al. (depending on fracture type: minimally invasive; open /closed reduction and internal fixation, bone cement application).

Osteological assessment included standardized laboratory measurements (e.g., serum 25(OH)-vitamin D level, calcium and phosphate levels, kidney values) and subsequent bone density evaluation (DEXA scan) during inpatient stay, if available.

Different assays for the assessment of serum 25(OH)-vitamin D level were used during the study period. The clinical medicine laboratory used the IDS RIA assay before 2010. Subsequently, from November 2010 to July 2015, the IDS CLIA assay was used. From July 2015 to September 2020, DiaSorin CLIA was used, and up to and including today, the Roche Diagnostics ECLIA Cobas assay was used. Vitamin D deficiency was defined as a serum 25(OH)-vitamin D of <20 ng/mL, vitamin D insufficiency as a serum-25(OH)-vitamin D of 21–29 ng/mL, and regular vitamin D levels as a serum-25(OH)-vitamin D of >30 ng/mL [26,27].

Bone density evaluation was performed by quantitative digital radiography in the past (QDR; QDR 2000/4000, Hologic, Waltham, MA, USA) and nowadays by dual-energy X-ray absorptiometry (DEXA) devices (Horizon DXA, Hologic, Waltham, MA, USA; Lunar iDXA, GE Healthcare, Madison, WI, USA). The study did not include patients whose T-score was derived from a qCT measurement to avoid bias of different measurement methods. Osteopenia was defined by a T-score from −1 to −2.5 standard deviations (SDs) and osteoporosis by a T-score <−2.5 SDs [28].

To assess regional bone density by Hounsfield units, CT scans with reconstructive images generated for pelvic ring fracture diagnostics were used. Computed tomography (CT) was performed by a trained radiologist according to a standardized protocol on the same machines (Somatom Definition Edge^®^, Siemens Healthengineers, Munich, Germany). The acquired CT images were processed using dedicated software (Visage Imaging GmbH, Visage 7, Berlin, Germany).

As previously described by other groups, we manually measured HUs in the reconstructive CT images by using various ROI (region of interest) circles drawn on defined points [17,18]. For each ROI, the largest possible circle, excluding the cortical bone, was placed. To ensure a standardized measurement process, the procedure described by Schönenberg et al. was followed. For each patient, HUs in the sacral body at level 1 were determined in the axial and sagittal plane, and in the sacral alae, only the axial plane was used [18]. Patients with transitional vertebra were included. Both in the axial and sagittal plane, the midpoint location of the S1 body and alae was defined by two cross-reference lines.

**Figure 6:**
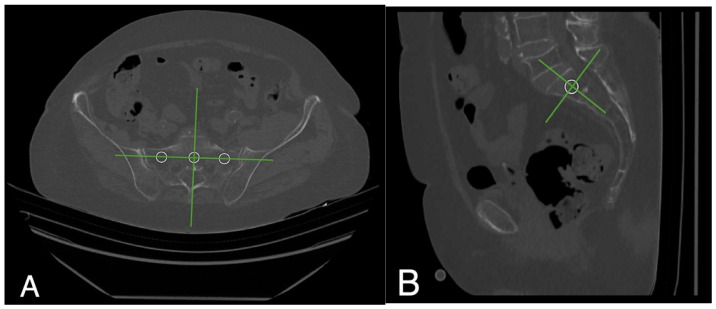
CT images of a 90-year-old female patient with a left-sided FFP IIc. (**A**) Axial view of the lateral fracture of the left massa lateralis; (**B**) sagittal view of the same fracture. The white circles represent the points to be measured at which the HU measurement is carried out. The green auxiliary lines are used for precise positioning.

In the sagittal plane, the first one is drawn parallel to the tangents of the inferior and superior vertebra and the second one to the most dorsal and ventral points of the sacral bodies. The midpoints of the spinal process and the tangent of the most anterior point of the sacral bodies were reference lines in the axial plane. Measuring HUs for the alae, lines were drawn through the midpoints of the sacral foramina as tangents to the most anterior point of the alae. If the fracture line passed through the defined measurement point, it was excluded from the determination of bone density using Hus (Figure 6).

Statistical analysis: Data are reported as either mean ± standard deviation (SD) or for categorical data as absolute frequency with a percentage distribution. The Kolmogorov–Smirnov test was used for ruling out normal distribution; subsequently, a Mann–Whitney U-test or t-test was used, while Fisher’s Exact Test was used for dichotome variables. For correlation analysis, the Pearson and Spearman correlation coefficients were used depending on the data distribution. A *p*-value < 0.05 was regarded to be statistically significant.

## 5. Conclusions

In summary, this study provides valuable insights into the osteological and diagnostic evaluation of FFPs in geriatric patients. The findings suggest that both CT-based HU values and DEXA-based T-scores offer important information regarding bone quality and fracture severity, although their clinical application should be considered with nuance. Based on our current presented results, the DEXA measurement remains the gold standard, even for FFPs. However, due to the commonly available CT scans, it is already possible to obtain initial key insights regarding bone health and bone density. Ideally, a specific therapy for potential osteoporosis can then already be initiated during the inpatient stay without any delay. Further studies are needed to clarify the precise relationship between vitamin D levels, bone density, and fracture types and to identify the best strategies for preventing and treating FFPs in older patients.

## Figures and Tables

**Table 1:** Baseline data.

Age, Mean ± SD	87.3 ± 4.5
Gender, *n* (%)	
male	93 (20.4)
female	363 (79.6)
CT based classification, *n* (%)	456 (100)
FFP Type, *n* (%)	
I	57 (12.5)
II	304 (66.7)
III	73 (16.0)
IV	22 (4.8)
Treatment, *n* (%)	
conservative	387 (84.9)
operative	69 (15.1)
Previously diagnosed osteoporosis, *n* (%)	67 (14.7)
Laboratory vitamin D measurement, *n* (%)	169 (37.1)
Vitamin D deficiency *, *n* (%)	106 (62.7)
(Serum 25(OH)-vitamin D	
level < 20 ng/mL)	
DEXA scan, *n* (%)	
lumbar vertebrae	99 (21.7)
femoral neck	99 (21.7)
Newly diagnosed osteopenia/osteoporosis *, *n* (%)	
T-Score −2.5–−1 in DEXA scan (osteopenia)	34 (34.3)
T-Score ≤ −2.5 in DEXA scan (osteoporosis)	46 (46.5)

* In assessed patients.

**Table 2:** Treatment regimens of different types of FFPs.

Total Patients	FFP Type I *n* = 57	FFP Type II *n* = 304	FFP Type III *n* = 73	FFP Type IV *n* = 22	*p*-Value
conservative, *n* (%)	56 (98.2)	272 (89.5)	50 (68.5)	9 (40.9)	
operative, *n* (%)	1 (1.8)	32 (10.5)	23 (31.5)	13 (59.1)	< 0.001

Correlation analysis with Spearman correlation coefficient.

**Table 3:** Osteoporosis/osteomalacia diagnostics in different types of FFPs.

Total Patients	FFP Type I *n* = 57	FFP Type II *n* = 304	FFP Type III *n* = 73	FFP Type IV *n* = 22	*p*-Value
Serum 25(OH)-vitamin D level ^1^ (ng/mL; mean ± SD)	*n* = 12	*n* = 112	*n* = 31	*n* = 14	
	17.67 ± 12.95	14.09 ± 15.09	16.89 ± 15.57	25.92 ± 11.57	0.042
T-score lumbar vertebrae (mean ± SD)	*n* = 10	*n* = 65	*n* = 18	*n* = 6	
	−1.76 ± 1.36	−2.07 ± 1.57	−2.69 ± 1.26	−2.80 ± 1.13	ns
T-score femoral neck (mean ± SD)	*n* = 10	*n* = 65	*n* = 18	*n* = 6	
	−2.19 ± 2.18	−2.59 ± 1.16	−2.57 ± 1.18	−2.80 ± 0.76	ns
Alkaline phosphatase ^2^ (U/L; mean ± SD)	*n* = 19 * 111.16 ± 46.37 *n* = 3 ° 161.33 ± 82.86	*n* = 135 * 107.16 ± 73.91 *n* = 31 ° 100.16 ± 44.19	*n* = 36 * 117.25 ± 112.36 *n* = 8 ° 99.50 ± 31.64	*n* = 14 * 150.57 ± 102.94 *n* = 2 ° 162.00 ± 69.30	ns ns
Serum calcium ^3^ (mmol/L; mean ± SD)	*n* = 18 2.30 ± 0.29	*n* = 149 2.36 ± 0.12	*n* = 38 2.38 ± 0.12	*n* = 13 2.39 ± 0.10	ns
Serum phosphate ^4^ (mg/dL; mean ± SD)	*n* = 18 3.30 ± 0.86	*n* = 148 3.15 ± 0.81	*n* = 38 3.13 ± 0.61	*n* = 13 3.13 ± 0.30	ns

* Female patients (range 35–105 U/L); ° male patients (range 40–130 U/L); ^1^ serum 25(OH)-vitamin D range 20–100 ng/mL; ^2^ alkaline phosphatase range 35–120 U/L; ^3^ serum calcium range 2.05–2.65 mmol/L; ^4^ serum phosphate range 2.5–4.8 mg/dL (correlation analysis with Spearman correlation coefficient).

**Table 4:** Baseline data of assessed Hounsfield units.

CT-Based HU, *n* (%)		251 (55.0)
Numbers of measured HU, *n* (%)		
Corpus S1		251 (100)
Ala S1		134 (53.4)
Ala S1 unilateral fracture		97 (38.6)
Ala S1 bilateral fracture		20 (8)
Hounsfield units (mean ± SD)		
Corpus S1 axial and sagittal		97.90 ± 3.17
Ala S1		−6.70 ± 3.15
Corpus S1 axial		91 ± 50
Corpus S1 sagittal		86 ± 51
Corpus S1	male axial	115.86 ± 53.94
	female axial	84.75 ± 53.63
Ala S1 without fracture	male	−6.24 ± 39.04
	female	−6.83 ± 34.99

**Table 5:** Hounsfield units in different types of FFPs.

	FFP Type I	FFP Type II	FFP Type III	FFP Type IV	*p*-Value ^
HU S1 axial	*n =* 27	*n =* 169	*n* = 42	*n* = 13	
	72.11 ± 53.00	96.30 ± 50.61	81.43 ± 45.97	88.85 ± 26.25	0.840
HU S1 sagittal	*n =* 27	*n =* 169	*n* = 42	*n* = 13	
	64.93 ± 55.40	90.57 ± 50.28	78.21 ± 51.11	88.92 ± 39.80	0.669
Ala sacrum S1 unfractured right	*n =* 23	*n =* 94	*n* = 11	*n* = 2	
	−32.96 ± 40.34	−19.12 ± 36.95	−30.45 ± 31.56	−28.00 ± 39.60	0.756
Ala sacrum S1 unfractured left	*n =* 23	*n =* 94	*n* = 11	*n* = 2	
	−21.43 ± 37.18	−16.05 ± 39.17	−36.27 ± 39.07	−9.00 ± 32.53	0.736
Ala sacrum S1 unfractured fracture	*n =* 4	*n =* 67	*n* = 28	*n* = 2	
	−22.50 ± 54.43	−28.27 ± 39.24	−34.43 ± 31.41	−33.50 ± 10.61	0.511
Ala sacrum S1 opposite side fractured	*n =* 4	*n =* 67	*n* = 28	*n* = 2	
	−12.75 ± 37.62	11.79 ± 40.56	4.89 ± 32.17	−5.50 ± 58.69	0.749
Ala sacrum S1 bilateral fractures	*n =* 0	*n =* 8	*n* = 3	*n* = 9	
		20.75 ± 37.70	31.67 ± 6.35	25.67 ± 45.87	0.748
Ala sacrum S1 opposite side bilateral fractures	*n =* 0	*n =* 8	*n* = 3	*n* = 9	
		29.88 ± 41.15	68.00 ± 17.52	26.00 ± 39.34	0.695

^ Correlation between HU values and fracture severity (correlation analysis by Pearson correlation coefficient).

**Table 6:** HUs and T-scores depending on vitamin D levels.

	Vitamin D Deficiency *	Regular Vitamin D Level	*p*-Value
T-score (mean ± SD)			
T-score L1–4	−2.2 ± 1.3	−2.6 ± 1.1	0.321
T-score femoral neck	−2.9 ± 0.5	−2.7 ± 0.9	0.681
Hounsfield units (mean *±* SD)			
Corpus S1 axial	90 ± 43	97 ± 34	0.262
Corpus S1 sagittal	91 ± 48	103 ± 36	0.453
Ala S1 axial without fracture right side	−37 ± 38	−17 ± 41	0.397
Ala S1 axial without fracture left side	−26 ± 33	−9 ± 30	0.397

* Vitamin D deficiency defined as a T-score ≤ −2.5 (correlation analysis by Spearman correlation coefficient).

**Table 7:** Distribution of HUs and T-scores depending on DEXA-based bone density.

	T-Scores ^1^	Hounsfield Units Level S1 ^2^
Regular	0.0 ± 1.3	113.33 ± 44.95
Osteopenia	−1.8 ± 0.4	87.85 ± 42.06
Osteoporosis	−3.0 ± 0.3	91.27 ± 38.14

^1^ Regular: T-score ≥ −1.0; osteopenia: T-score between −1.5 and −2.5; osteoporosis: T-score ≤ −2.5; mean T-scores from lumbar vertebrae and femoral neck; ^2^ mean HU from corpus S1 in axial and sagittal plane.

## Data Availability

Data are available from the corresponding author upon reasonable request.
